# Growth Differentiation Factor-15 as a Predictor of Idiopathic Membranous Nephropathy Progression: A Retrospective Study

**DOI:** 10.1155/2018/1463940

**Published:** 2018-02-22

**Authors:** Young Rok Ham, Chang Hun Song, Hong Jin Bae, Jin Young Jeong, Min-Kyung Yeo, Dae Eun Choi, Ki-Ryang Na, Kang Wook Lee

**Affiliations:** ^1^Division of Nephrology, Department of Internal Medicine, Chungnam National University Hospital, Daejeon, Republic of Korea; ^2^Department of Medical Science, Chungnam National University, Daejeon, Republic of Korea; ^3^Department of Pathology, Chungnam National University School of Medicine, Daejeon, Republic of Korea

## Abstract

Idiopathic membranous nephropathy (IMN) is a major cause of nephrotic syndrome. No biomarker to predict the long-term prognosis of IMN is currently available. Growth differentiation factor-15 (GDF-15) is a member of the transforming growth factor-*β* superfamily and has been associated with chronic inflammatory disease. It has the potential to be a useful prognostic marker in patients with renal diseases, such as diabetic nephropathy and IgA nephropathy. This study examined whether GDF-15 is associated with the clinical parameters in IMN and showed that GDF-15 can predict IMN disease progression. A total of 35 patients with biopsy-proven IMN, treated at Chungnam National University Hospital from January 2010 to December 2015, were included. Patients younger than 18 years, those with secondary membranous nephropathy, and those lost to follow-up before 12 months were excluded. Levels of GDF-15 at the time of biopsy were measured using enzyme-linked immunosorbent assays. Disease progression was defined as a ≥30% decline in estimated glomerular filtration rate (eGFR) or the development of end-stage renal disease. The mean follow-up was 44.1 months (range: 16–72 months). Using receiver operating curve analysis, the best serum GDF-15 cut-off value for predicting disease progression was 2.15 ng/ml (sensitivity: 75.0%, specificity: 82.1%, *p* = 0.007). GDF-15 was significantly related to age and initial renal function. In the Kaplan-Meier analysis, the risk of disease progression increased in patients with GDF-15 ≥ 2.15 ng/ml when compared with those with GDF-15 < 2.15 ng/ml (50.0% versus 9.7%) (*p* = 0.012). In the multivariate Cox regression analysis adjusted for potential confounders, only GDF-15 was significantly associated with disease progression in IMN (*p* = 0.032). In conclusion, the GDF-15 level at the time of diagnosis has a significant negative correlation with initial renal function and is associated with a poor prognosis in IMN. Our results suggest that GDF-15 provides useful prognostic information in patients with IMN.

## 1. Introduction

Idiopathic membranous nephropathy (IMN) represents approximately 20–30% of all cases of the nephrotic syndrome in adults [1, 2] and accounts for 70–80% of all patients with membranous nephropathy (MN) [1, 3]. The clinical features and prognosis of IMN are variable, ranging from spontaneous remission of nephrotic syndrome (up to 20–60%) to a slow, progressive decline in glomerular filtration rate over several years [4–6]. It still remains unclear when and which immunosuppressive treatment should be used. Persistent heavy proteinuria, a steep decline in creatinine clearance, initial impairment of renal function, severe interstitial fibrosis, and the presence of autoantibodies against phospholipase A_2_ receptor (anti-PLA_2_R) are known risk factors for renal disease progression [7–10]. These markers are nonetheless unable to fully predict the diverse clinical course of IMN. Therefore, precise biomarkers for the prediction of renal disease progression and treatment response are needed.

Recently, it was found that expression of growth differentiation factor-15 (GDF-15) in conjunction with other inflammatory markers is upregulated by oxidative stress, tissue ischemia, and cancer in organ tissues, including the heart and kidneys [11, 12]. Previous studies suggested that GDF-15 might be a risk factor and prognostic biomarker for cardiovascular disease [13–15]. Recently, there has been increasing interest in the relationship between GDF-15 and kidney disease in diseases such as diabetes and IgA nephropathy [16, 17]. Lajer et al. reported that elevated circulating GDF-15 was significantly associated with a worse renal outcome in insulin-dependent diabetic patients with macroalbuminuria [16]. Our previous work in patients with IgA nephropathy showed that serum GDF-15 is significantly associated with adverse renal function and histologic findings [17]. This led us to hypothesize that GDF-15 may play a role in the pathogenesis of inflammatory kidney disease; however, no work has examined the association between GDF-15 and clinical manifestations in patients with IMN.

We hypothesize that an elevated level of GDF-15 is associated with initial worsening of IMN and plays a role in disease progression in IMN. Therefore, this study investigated whether GDF-15 is associated with the clinical parameters in IMN and determined whether GDF-15 is associated with IMN prognosis through a comparison of parameters, including proteinuria, initial impairment of renal function, interstitial fibrosis, and PLA_2_R Ab.

## 2. Material and Methods

### 2.1. Study Population

A total of 64 patients with biopsy-proven MN, retreated at Chungnam National University Hospital from January 2010 to December 2015, were evaluated for inclusion in this retrospective analysis, of whom 18 were excluded for nonavailability of urine or plasma samples. Of the remaining 46 patients, patients younger than 18 years (*n* = 1), those with secondary MN (*n* = 7), those with previous kidney transplantation (*n* = 1), and those who were followed up for less than 12 months (*n* = 3) were excluded. A total of 35 patients (24 males: 68.6%, mean age: 59.9 ± 13.2 years) with previously diagnosed IMN were included in the study. This study was reviewed and approved by the Ethics Committee of Chungnam National University Hospital and was conducted in accordance with the guidelines of the Declaration of Helsinki.

### 2.2. Clinical Parameters

Baseline data at the time of renal biopsy were obtained from medical records and included age, sex, the presence of hypertension (HTN) or diabetes mellitus (DM), and the prescription of nephrotoxic drugs, such as nonsteroidal anti-inflammatory drugs (NSAIDs) or aminoglycosides. Laboratory data for serum creatinine, albumin, estimated glomerular filtration rate (eGFR), and the spot urine protein-to-creatinine ratio (UPCR) were obtained in the morning after admission under fasting conditions. The eGFR was calculated using the Modification in Diet and Renal Disease (MDRD) equation. Treatment data included the prescription of immunosuppressive agents, such as steroids, cyclophosphamide, cyclosporine, and mycophenolate mofetil, as well as antihypertensive medications including angiotensin-converting-enzyme inhibitors (ACEis) and/or angiotensin receptor blockers (ARBs).

### 2.3. Measurement of Serum GDF-15 and PLA_2_R Ab Levels

Blood samples for the detection of GDF-15 were collected in the morning after admission under fasting conditions. Blood samples were centrifuged immediately after collection and were stored at −70°C before use. Serum GDF-15 concentrations were measured with quantitative enzyme-linked immunosorbent assay (ELISA) kits (R&D Systems, Minneapolis, MN, USA). Serum anti-PLA_2_R antibody concentrations were measured by a commercial ELISA kit (LSBio, Seattle, WA, USA) according to the manufacturer's instructions. The intra-assay and interassay coefficients of variation were less than 10% and 12%, respectively. The mean values of duplicate results were used for the analysis.

### 2.4. Study Group Design and End Points

Patients were divided into two groups depending on their serum GDF-15 levels at the time of renal biopsy. GDF-15 levels were <2.15 ng/ml in group 1 (lower GDF-15 levels) and ≥2.15 ng/ml in group 2. Disease progression was defined as a ≥30% decline in baseline eGFR, having being diagnosed with end-stage renal disease requiring renal replacement therapy after follow-up [18].

### 2.5. Histologic Evaluation

All renal biopsy tissues acquired at the time of initial diagnosis were reviewed and classified in a semiquantitative manner by an experienced pathologist. The pathologist was blinded to the patient's details to reduce observer bias. Optical microscopy findings regarding the glomeruli, tubules, and interstitium were scored with respect to the severity of the lesions. Glomerular sclerosis was categorized according to the proportion of glomerular sclerosis, as follows: G1, 0–25% and G2, >25% [19]. Interstitial fibrosis/tubular atrophy (IF/TA) was classified as T0 when <15% of IF/TA were involved and T1 when ≥15% of IF/TA were involved [20].

### 2.6. Statistical Analysis

A comparison of univariate predictors of clinical outcomes between groups was performed using *χ* ^2^ tests for categorical variables and the Kruskal-Wallis or Mann–Whitney test for continuous variables. Differences in continuous variables between the two groups were assessed using independent *t*-tests. Continuous variables are expressed as means ± SD, and categorical variables are expressed as frequencies and percentages. Receiver operating characteristic (ROC) curve analysis was performed to calculate the area under the curve (AUC) for GDF-15, PLA_2_R Ab, eGFR, and UPCR to determine the best cut-off value to predict renal progression. The renal progression-free rates were calculated using Kaplan-Meier analysis, and comparisons between groups were performed using the log-rank test. Multivariate Cox proportional hazards regression analysis was performed to determine independent variables associated with renal outcomes. All statistical analyses were performed using SPSS for Windows software (ver. 21.0; IBM Corp., Armonk, NY, USA). A *p* value < 0.05 was considered to represent statistical significance.

## 3. Results

### 3.1. Prediction of Renal Disease Progression Using GDF-15 Levels

**Figure 1:**
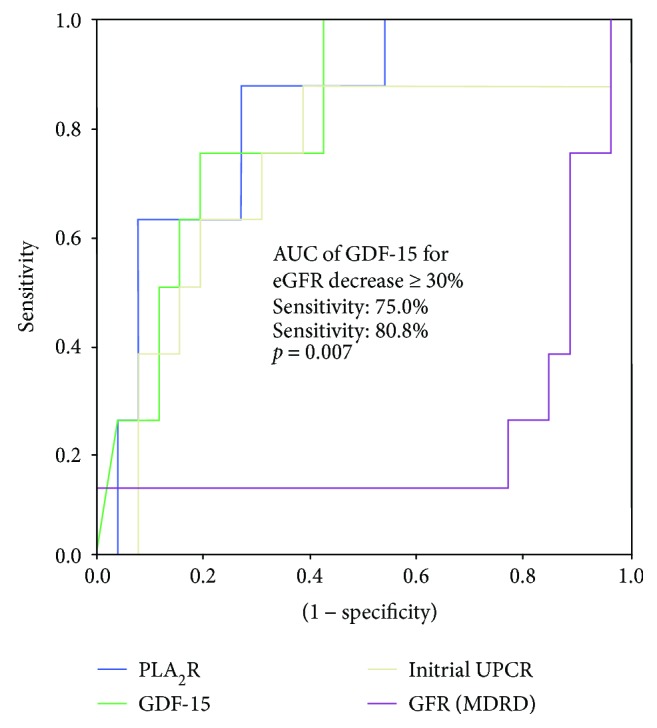
ROC curve and performance in predicting renal progression. ROC curve showing the prognostic sensitivity and specificity of GDF-15, PLA_2_R Ab, UPCR, and initial eGFR with regard to renal progression. GDF-15 > 2.15 ng/ml demonstrates 80.8% specificity and 75.0% sensitivity in predicting disease progression. ROC: receiver operating characteristic; GDF: growth differentiation factor (green line); PLA_2_R Ab: phospholipase A_2_ receptor antibody (blue line); UPCR: spot urine protein-to-creatinine ratio (yellow line); eGFR: estimated glomerular filtration rate (violet line); AUC: area under the receiver operating characteristic curve.

The ROC curve analysis of GDF-15, PLA_2_R Ab, eGFR, and UPCR for the determination of disease progression is illustrated in Figure 1. The AUCs for GDF-15, PLA_2_R Ab, UPCR, and eGFR were 0.817 (95% confidence interval [95% CI]: 0.669–0.966, *p* = 0.007), 0.827 (95% CI: 0.675–0.979, *p* = 0.006), 0.721 (95% CI: 0.501–0.942, *p* = 0.062), and 0.226 (95% CI: 0.000–0.452, *p* = 0.021), respectively. The best cut-off value for GDF-15 and PLA_2_R Ab for predicting disease progression was 2.15 ng/ml (sensitivity: 75.0%, specificity: 82.1%) and 2.77 ng/ml (sensitivity: 87.5%, specificity: 73.1%), respectively.

### 3.2. Clinical Baseline Characteristics

The baseline characteristics of the study patients are shown in Table 1. The mean follow-up period was 44.1 months (range: 16–72 months). Most patients (28, 80.0%) showed preserved renal function (eGFR ≥ 60 ml/min per 1.73 m^2^), and 19 patients (54.3%) had nephrotic range proteinuria. The number of diabetic and hypertensive patients was 6 (17.1%) and 18 (51.4%), respectively. Of the patients, 27 (77.1%) received ACEi or ARB and 21 (60.0%) were prescribed immunosuppressant agents (Supplement Table 1) and only 2 patients received NSAIDs; however, after the diagnosis of IMN, they discontinued the NSAIDs. None of the patients in the study had coronary artery disease and heart failure, whereas one patient developed venous thrombosis. By the end of the observation period, three patients (8.6%) reached end-stage renal disease and were maintained on renal replacement therapy.

### 3.3. Association of Serum GDF-15 Levels with Clinical and Biochemical Parameters

To assess whether GDF-15 is related to disease severity, we investigated the relationship between GDF-15 levels and clinical parameters. The mean serum GDF-15 concentration in all patients was 1.73 ± 0.77 ng/ml (range: 0.32–2.97 ng/ml). The association between GDF-15 levels and clinical parameters is shown in Table 1. The patients in group 2 were older (*p* = 0.012) and more likely to have DM at baseline (*p* = 0.001) than those in group 1. Serum hemoglobin and eGFR were lower (*p* = 0.012, *p* = 0.009, resp.) in group 2 than in group 1. In contrast, serum creatinine, PLA_2_R Ab, and IF/TA (%) were higher in group 2 than in group 1 (*p* = 0.049, *p* = 0.019, and *p* = 0.025, resp.). No significant difference was observed in HTN, body mass index (BMI), UPCR, serum albumin, total cholesterol, or glomerular sclerosis (Table 1) between the two groups.

**Figure 2:**
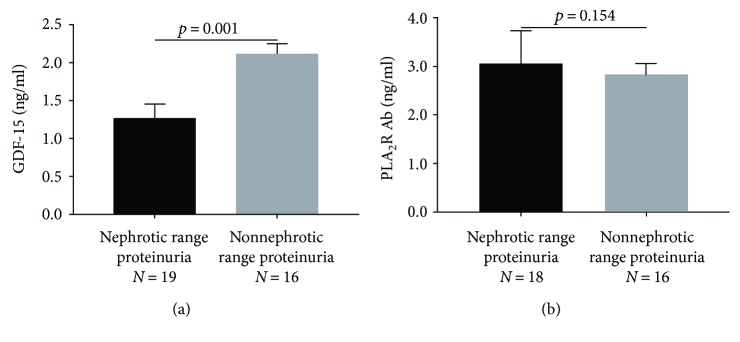
Relationship between serum GDF-15 level and proteinuria and serum PLA_2_R Ab level and proteinuria. Distribution of serum GDF-15 in patients stratified by (a) nephrotic range and nonnephrotic proteinuria. Distribution of serum PLA_2_R Ab in patients stratified by (b) nephrotic range and nonnephrotic proteinuria. GDF: growth differentiation factor; PLA_2_R Ab: phospholipase A_2_ receptor antibody; eGFR: estimated glomerular filtration rate.

Patients with nephrotic range proteinuria had higher GDF-15 levels than those with nonnephrotic proteinuria (2.11 ± 0.59 versus 1.27 ± 0.72 ng/ml, *p* = 0.001, Figure 2(a)). By contrast, there was no significant difference in PLA_2_R Ab levels according to proteinuria (*p* = 0.154, Figure 2(b)).

**Figure 3:**
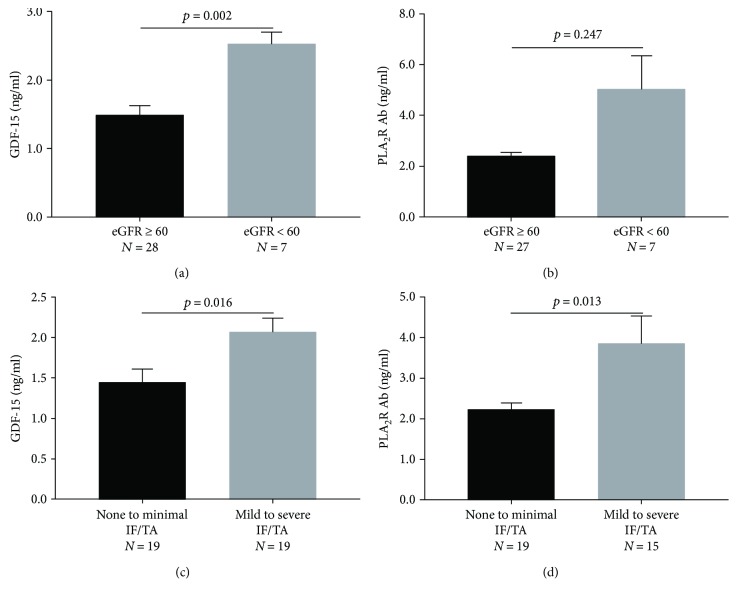
Relationship between serum GDF-15 level and eGFR and IF/TA and serum PLA_2_R Ab level and eGFR and IF/TA. Distribution of serum GDF-15 in patients stratified by (a) eGFR ≥ 60 and eGFR < 60 and (c) none to minimal IF/TA and mild to severe IF/TA. Distribution of serum PLA_2_R Ab in patients stratified by (b) eGFR ≥ 60 and eGFR < 60 and (d) none to minimal IF/TA and mild to severe IF/TA. GDF: growth differentiation factor; PLA_2_R Ab: phospholipase A_2_ receptor antibody; eGFR: estimated glomerular filtration rate; IF/TA: interstitial fibrosis/tubular atrophy.

Patients with decreased renal function (eGFR < 60 ml/min per 1.73 m^2^) had elevated levels of GDF-15 compared with patient with preserved renal function (eGFR ≥ 60 ml/min per 1.73 m^2^) (2.46 ± 0.49 versus 1.54 ± 0.72 ng/ml, *p* = 0.002, Figure 3(a), Supplement Table 2) at the time of diagnosis. However, there was no significant difference in PLA_2_R Ab level according to eGFR (*p* = 0.247, Figure 3(c)). In multivariate regression analysis, only GDF-15 was significantly associated with decreased renal function at the time of diagnosis (OR 17.387, 95% CI: 1.232–245.286, *p* = 0.034, Table 2).

### 3.4. Serum GDF-15 Levels and Histologic Findings

There was no significant difference in GDF-15 level according to the degree of glomerular sclerosis. In IF/TA, patients at stage T1 had elevated GDF-15 (*p* = 0.016, Figure 3(c)) and PLA_2_R Ab (*p* = 0.013, Figure 3(d)) levels compared with those at stage T0 (Supplement Table 3) in univariate analyses. However, in multivariate regression analysis, only PLA_2_R Ab was significantly associated with initial severe IF/TA (OR 10.147, 95% CI: 1.223–84.170, *p* = 0.032, Table 2).

### 3.5. Disease Progression and Clinical Parameters in IMN

We evaluated the association between renal outcomes and clinical parameters. Patients were treated according to the Kidney Disease Improving Global Outcomes (KDIGO) guidelines [21]. A total of 27 patients (77.1%) were treated with renin-angiotensin-inhibiting agents, 23 (65.7%) with statins, and 21 (60.0%) with immunosuppressive agents. Eight patients (22.9%) experienced disease progression (≥30% decline in eGFR or the initiation of renal replacement therapy) during the follow-up period. Patients were divided into two groups (progression and nonprogression groups) according to whether renal disease had progressed. In the progression group, GDF-15, PLA_2_R Ab, and IF/TA were significantly higher, while eGFR was lower, than in the nonprogression group; no other parameters showed statistically significant differences (Table 3).

### 3.6. High Serum GDF-15 Is Associated with Disease Progression in IMN

**Figure 4:**
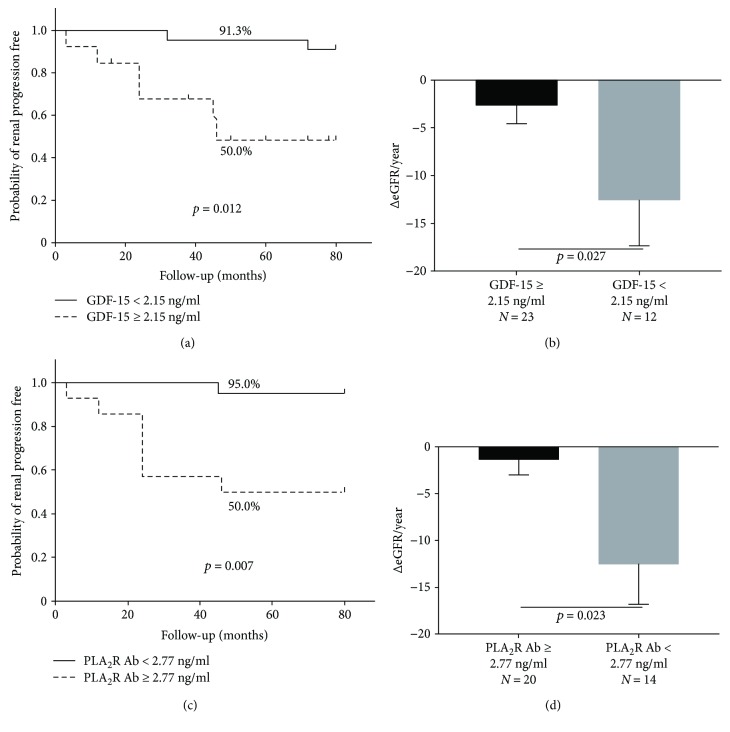
Kaplan-Meier analysis of renal survival on GDF-15 and PLA_2_R Ab. Kaplan-Meier curve (a) for the occurrence of disease progression (log-rank *p* = 0.012) showed a significant difference between groups 1 and 2. (b) Patients in group 2 showed higher ΔeGFR/year than patients in group 1 (*p* = 0.027). Kaplan-Meier curve (c) for the occurrence of disease progression (log-rank *p* = 0.007) also showed a significant difference between patients with PLA_2_R Ab ≥ 2.77 ng/ml and patients with PLA_2_R Ab < 2.77 ng/ml. (d) Patients with PLA_2_R Ab ≥ 2.77 (ng/ml) showed higher ΔeGFR/year than patients with PLA_2_R Ab < 2.77 (ng/ml) (*p* = 0.023). GDF: growth differentiation factor; slope of ΔeGFR: (final eGFR − initial eGFR)/6; PLA_2_R Ab: phospholipase A_2_ receptor antibody.

Disease progression was more significant in group 2 than in group 1 according to Kaplan-Meier survival analysis (50.0% versus 8.7%, *p* = 0.012, Figure 4(a)). Patients with high PLA_2_R Ab (≥2.77 ng/ml) showed a significantly higher rate of disease progression than those with low PLA_2_R Ab (<2.77 ng/ml) (50.0% versus 5.0%, *p* = 0.007, Figure 4(c)). We assessed the annual eGFR decrease (ΔeGFR/year) in all patients. Patients with high GDF-15 and PLA_2_R Ab showed a significantly increased decline in eGFR versus those with low GDF-15 and PLA_2_R Ab (*p* = 0.027, *p* = 0.023, Figures 4(b) and 4(d), resp.).

All confounding variables, including age, the presence of DM, eGFR, UPCR, PLA_2_R Ab, pathologic state (IF/TA), and GDF-15, were included in simple Cox regression analysis to determine the independent effect of GDF-15 on disease progression (Table 4). GDF-15, PLA_2_R Ab, UPCR, and IF/TA were significantly associated with disease progression in univariate analysis. In multivariate Cox proportional analysis, GDF-15 (≥2.15 ng/ml) remained independently associated with disease progression after adjusting for confounding variables, including age, male gender, the presence of DM or HTN, PLA_2_R Ab, eGFR, UPCR, and IF/TA. The patients in group 2 were more than thirty-three times more likely than those in group 1 to show disease progression (hazard ratio [HR]: 33.161 (1.341–820.034), *p* = 0.032).

## 4. Discussion

We found that elevated GDF-15 (≥2.15 ng/ml) was independently associated with a high risk of renal disease progression as defined as a 30% decline in eGFR or the development of ESRD, even after adjusting for additional risk factors in IMN. We also showed that circulating GDF-15 levels at the time of renal biopsy had a significantly negative correlation with initial renal function in IMN. Previous studies demonstrated that high circulating GDF-15 levels had a significant correlation with a faster decline of renal function in patients with type 1 DM, IgA nephropathy, and CKD stages 1–4 [16–18]. Moreover, studies of community dwelling elderly individuals reported a change of GDF-15 concentration related not only to the baseline renal function but also to the change in renal function over the 5-year study period [22]. Our study extends the findings of these previous studies in that GDF-15 levels at the time of diagnosis are associated with initial renal function and could predict the risk of disease progression in patients with IMN.

GDF-15 was shown to be involved in promoting anti-inflammatory pathways in several pathologic conditions including inflammation, cancer, cardiovascular disease, and pulmonary disease [23–26]. In terms of the kidneys, preclinical studies in diabetic mice [27] and in mice with septic renal injured mice [28] showed that GDF-15 KO mice had increased interstitial and tubular damage without direct glomerular damage and augmented inflammation, respectively; administration of GDF-15 ameliorates apoptosis and inflammation [28]. Another study observed that increased expression of GDF1-15 was induced in a mouse model by renal injury, such as caused by the injection of carbon tetrachloride (CCl_4_), 5/6 nephrectomy, and ischemia-reperfusion injury [11]; additionally, increased urinary GDF-15 was associated with proximal tubular damage in *db*/*db* mice [29]. In a study with CKD patients, plasma GDF-15 had a significant positive correlation with expression of GDF-15 mRNA in the kidneys [18]. Taking together, these results suggest renal injury may increase serum GDF-15, and elevated GDF-15 protects against renal damage and indicates more severe renal injury. Although GDF-15 protects against renal injury, a high GDF-15 is associated with severe renal injury and a poor prognosis of renal disease, as also seen in the heart [30, 31]. Further studies need to identify whether the administration of GDF-15 can reduce the renal injury and improve the prognosis in IMN.

PLA_2_R Ab is mainly expressed in podocytes and has been known to act as a major antigenic target involved in IMN [32], and a significant association between antibody titer and disease activity and progression in IMN was reported in previous studies [20, 33]. In our study, PLA_2_R Ab showed a positive correlation with glomerular sclerosis and GDF-15. The patients with high PLA_2_R Ab showed higher rates of disease progression than patients with low PLA_2_R Ab in univariate analysis. However, PLA_2_R Ab could not be an independent predictor for disease progression after controlling for other parameters. This suggested that GDF-15 is more closely related to the prognosis of IMN than PLA_2_R Ab.

The relationship between elevated GDF-15 and abnormal glucose control is well documented in previous studies [34, 35]. Our study also showed that elevated GDF-15 levels were significantly associated with the presence of DM. To avoid statistical bias for determining the prognostic impact of DM, we excluded patients who showed diabetic nephropathy on renal biopsy. Therefore, we believe that the association between GDF-15 and the prognosis of IMN is independent of DM.

Based on these results, we propose that elevated GDF-15 is significantly associated with a poor prognosis in IMN. However, the underlying mechanisms of elevated GDF-15 leading to increased disease progression remain unknown. We suggested that high GDF-15 reflects advanced renal injury and a high degree of proteinuria in IMN and that both these conditions cause a rapid disease progression. Further studies are needed to identify the exact mechanisms of GDF-15 in IMN.

Our study has some limitations. First, our study has a very small number of IMN patients that strongly limit the statistical power. Second, a retrospective design to the study could not exclude all confounding factors. Third, we were unable to measure intrarenal GDF-15 expression. However, other studies demonstrated that intrarenal GDF-15 is directly reflective of circulating GDF-15 [18]. Fourth, follow-up samples were not collected and therefore changes to GDF-15 levels at follow-up could not be demonstrated. More studies are needed to monitor how GDF-15 levels change after treatment.

In conclusion, the GDF-15 levels at the time of diagnosis have a significant negative correlation with the initial renal function and a significant positive correlation with a higher risk of disease progression in IMN. Our results suggest that GDF-15 provides useful prognostic information in patients with IMN.

## Figures and Tables

**Table 1:** Baseline characteristics of the study subjects.

	All (*N* = 35)	Group 1 (*N* = 23) GDF-15 < 2.15 ng/ml	Group 2 (*N* = 12) GDF-15 ≥ 2.15 ng/ml	*p* value
Age (years)	59.9 ± 13.2 (24–80)	56.0 ± 12.8	67.6 ± 10.9	0.012
Male sex, *n* (%)	24	15 (65.2%)	9 (75.0%)	0.424
BMI (kg/m^2^)	24.2 ± 3.3 (18.0–31.8)	24.1 ± 3.2	24.3 ± 3.66	0.887
Systolic BP (mmHg)	130.2 ± 22.6 (98–192)	128.9 ± 23.6	131.7 ± 21.3	0.641
Diastolic BP (mmHg)	77.8 ± 12.8 (55–107)	78.4 ± 12.6	76.7 ± 13.7	0.731
DM, *n* (%)	6 (17.1%)	0 (0.0%)	6 (50.0%)	0.001
HTN, *n* (%)	18 (51.4%)	10 (43.5%)	8 (66.7%)	0.172
ACEi or ARB, *n* (%)	27 (77.1%)	18 (78.3%)	9 (75.0%)	0.571
*Laboratory parameters*				
Hemoglobin (g/dl)	12.9 ± 1.6 (8.6–16.1)	13.5 ± 1.5	11.9 ± 1.3	0.012
Serum albumin (g/dl)	2.86 ± 0.73 (1.6–4.4)	2.95 ± 0.79	2.69 ± 0.60	0.327
Serum creatinine (mg/dl)	1.05 ± 0.51 (0.5–2.5)	0.90 ± 0.37	1.33 ± 0.63	0.049
eGFR (ml/min per 1.73 m^2^)	83.6 ± 28.7 (26.2–126.8)	92.5 ± 22.4	66.1 ± 32.6	0.009
Glucose (mg/dl)	98.7 ± 29.5 (50–194)	91.9 ± 23.0	111.8 ± 36.7	0.056
Total cholesterol (mg/dl)	277.8 ± 97.5 (143–594)	292.3 ± 103.7	246.1 ± 77.5	0.202
LDL cholesterol (mg/dl)	172.8 ± 62.8 (68–302)	187.7 ± 64.2	139.9 ± 47.2	0.056
UPCR (g/g Cr)	4.5 ± 4.2(0.05–15.26)	3.64 ± 3.98	6.15 ± 4.36	0.096
GDF-15 (pg/ml)	1.73 ± 0.77 (0.32–2.97)	1.28 ± 0.50	2.59 ± 0.31	0.000
PLA_2_R Ab (ng/ml)	2.76 ± 1.14 (1.30–6.10)	2.37 ± 0.75	3.58 ± 1.41	0.019
*Histologic parameters*				
Sclerosis (>25%), *n* (%)	12 (34.3%)	8 (34.8%)	4 (33.3%)	0.618
IF/TA (%)	12.2 ± 7.98	10.0 ± 7.1	16.3 ± 8.3	0.025
IF/TA (≥15%), *n* (%)	16 (45.7%)	8 (34.8%)	8 (66.7%)	0.075

BMI: body mass index; BP: blood pressure; DM: diabetes mellitus; HTN: hypertension; ACEi or ARB: angiotensin-converting-enzyme inhibitor or angiotensin receptor blocker; eGFR: estimated glomerular filtration rate; UPCR: spot urine protein-to-creatinine ratio; GDF-15: growth differentiation factor-15; PLA_2_R Ab: phospholipase A_2_ receptor antibody; IF/TA: interstitial fibrosis/tubular atrophy.

**Table 2:** Multivariate regression analysis of initial renal function and interstitial fibrosis/tubular atrophy.

Factors	Initial renal function (eGFR <60)		IF/TA (≥15%)	
	OR (95% CI)	*p* value	OR (95% CI)	*p* value
GDF-15 (≥2.15 ng/ml)	17.387 (1.232–245.286)	0.034	1.367 (0.157–11.909)	0.777
PLA_2_R Ab (≥2.77 ng/ml)	1.222 (0.066–22.483)	0.037	10.147 (1.223–84.170)	0.032
eGFR			0.981 (0.946–1.018)	0.303
IF/TA (≥15%)	0.190 (0.012–2.935)	0.125		
Sclerosis (≥25%)	1.395 (0.083–23.559)	0.076	1.214 (0.149–9.916)	0.856

eGFR: estimated glomerular filtration rate; OR: odd ratio; CI: confidence interval; GDF-15: growth differentiation factor-15; PLA_2_R Ab: phospholipase A_2_ receptor antibody; IF/TA: interstitial fibrosis/tubular atrophy.

**Table 3:** Comparison of the clinical characteristics with respect to disease progression.

Variable (*N* = 35)	Nonprogression (*N* = 27)	Progression (*N* = 8)	*p* value
Age (years)	59.2 ± 13.0	62.6 ± 14.5	0.527
Diabetes mellitus, *n* (%)	4 (14.8%)	2 (25.0%)	0.420
Hypertension, *n* (%)	12 (44.4%)	6 (75.0%)	0.132
ACEi or ARB, *n* (%)	21 (77.8%)	6 (75.0%)	0.604
Serum albumin (g/dl)	2.9 ± 0.8	2.8 ± 0.6	0.788
Serum creatinine (mg/dl)	0.95 ± 0.41	1.39 ± 0.69	0.116
eGFR (ml/min per 1.73 m^2^)	89.5 ± 25.9	63.9 ± 30.4	0.024
Slope of ΔeGFR during 6 months	0.003 ± 0.041	−0.059 ± 0.1096	0.156
UPCR (g/g Cr)	3.8 ± 3.9	6.7 ± 4.6	0.089
Serum GDF-15 (ng/ml)	1.54 ± 0.73	2.36 ± 0.53	0.006
Serum PLA_2_R Ab (ng/ml)	2.49 ± 1.07	3.61 ± 0.98	0.013
Glomerular sclerosis (%)	19.2 ± 19.8	24.5 ± 29.4	0.553
IF/TA (%)	10.4 ± 6.6	18.1 ± 9.6	0.013
Glomerular sclerosis (≥25%), *n* (%)	8 (34.8%)	4 (33.3%)	0.618
IF/TA (≥15%), *n* (%)	10 (37.0%)	6 (75.0%)	0.068

DM: diabetes mellitus; HTN: hypertension; ACEi or ARB: angiotensin-converting-enzyme inhibitor or angiotensin receptor blocker; eGFR: estimated glomerular filtration rate; slope of ΔeGFR: (final eGFR − initial eGFR)/6; UPCR: spot urine protein-to-creatinine ratio; GDF: growth differentiation factor; PLA_2_R Ab: phospholipase A_2_ receptor antibody; IF/TA: interstitial fibrosis/tubular atrophy.

**Table 4:** Multivariate analysis for the occurrence of renal progression.

Factors	Univariate		Multivariate	
	HR (95% CI)	*p* value	HR (95% CI)	*p* value
Group 1 (reference)	1.000		1.000	
Group 2	5.896 (1.185–29.337)	0.030	33.161 (1.341–820.034)	0.032
Age (years)	1.029 (0.965–1.097)	0.383	0.976 (0.879–1.083)	0.588
Male	1.636 (0.329–8.126)	0.547	0.679 (0.013–34.270)	0.847
Diabetes mellitus	0.721 (0.144–3.604)	0.690	5.023 (0.093–272.503)	0.428
Hypertension	0.306 (0.062–1.519)	0.148	0.059 (0.001–2.986)	0.157
eGFR (mL/min/1.73 m^2^)	0.974 (0.949–1.000)	0.052	0.969 (0.915–1.027)	0.233
Serum PLA_2_R Ab (≥2.77 ng/ml)	9.876 (1.21–80.46)	0.032	13.152 (0.884–195.703)	0.055
UPCR (g/g Cr)	1.152 (1.006–1.320)	0.040	1.278 (0.945–1.727)	0.111
IF/TA (%)	1.091 (1.007–1.182)	0.032	0.913 (0.768–1.086)	0.304
Glomerular sclerosis (%)	1.014 (0.985–1.044)	0.343		
ACEi or ARB	0.785 (0.158–3.899)	0.767		

GDF: growth differentiation factor; HR: hazard ratio; CI: confidence interval; eGFR: estimated glomerular filtration rate; PLA_2_R Ab: phospholipase A_2_ receptor antibody; UPCR: spot urine protein-to-creatinine ratio; IF/TA: interstitial fibrosis/tubular atrophy; ACEi: angiotensin-converting-enzyme inhibitor; ARB: angiotensin receptor blocker.

## Supplementary Materials

Supplementary Materials Supplement Table 1: kind and dose of ACEi or ARB. Supplement Table 2: clinical characteristics by eGFR status. Patients with decreased renal function (eGFR < 60 ml/min per 1.73 m^2^) had elevated levels of GDF-15 and IF/TA compared with patient with preserved renal function (eGFR ≥ 60 ml/min per 1.73 m^2^) (2.46 ± 0.49 versus 1.54 ± 0.72 ng/ml, *p* = 0.003, *p* = 0.036, resp.) at the time of diagnosis. Supplement Table 3: clinical characteristics by IF/TA status. Patients at stage T1 had elevated GDF-15 (*p* = 0.016) and PLA_2_R Ab (*p* = 0.001) levels and decreased eGFR (*p* = 0.005) compared with those at stage T0.
