# Supplementary material for: Growth Differentiation Factor-15 as a Predictor of Idiopathic Membranous Nephropathy Progression: A Retrospective Study
Source: Dis Markers. 2018 Feb 22;2018:1463940. doi: 10.1155/2018/1463940 (PMC5842742; doi:10.1155/2018/1463940)
Supplement: Supplementary Materials — Supplement Table 1: kind and dose of ACEi or ARB. Supplement Table 2: clinical characteristics by eGFR status. Patients with decreased renal function (eGFR < 60 ml/min per 1.73 m2) had elevated levels of GDF-15 and IF/TA compared with patient with preserved renal function (eGFR ≥ 60 ml/min per 1.73 m2) (2.46 ± 0.49 versus 1.54 ± 0.72 ng/ml, p = 0.003, p = 0.036, resp.) at the time of diagnosis. Supplement Table 3: clinical characteristics by IF/TA status. Patients at stage T1 had elevated GDF-15 (p = 0.016) and PLA2R Ab (p = 0.001) levels and decreased eGFR (p = 0.005) compared with those at stage T0. [file 1463940.f1.docx]

**Supplement Table 1.** Kind and dose of ACEi or ARB.

| **Kind** | **Dose** | **Non-progression : progression (n)** |
| --- | --- | --- |
| Candesartan | 4mg  8mg  32mg | 2:0  4:0  0:1 |
| Irbesartan | 75mg  150mg | 0:1  3:0 |
| Olmesartan | 20mg | 1:3 |
| Losartan | 50mg | 4:0 |
| Telmisartan | 40mg  80mg | 4:0  1:0 |
| Valsartan | 40mg | 1:0 |
| Fimasartan | 60mg  120mg | 0:1  1:0 |

**Supplement table 2.** Clinical characteristics by eGFR status

| **Variables (N = 35)** | **eGFR ≥ 60 (N=28)** | **eGFR < 60 (N=7)** | ***P* value** |
| --- | --- | --- | --- |
| Age (years) | 58.9±12.9 | 64.3±14.7 | 0.343 |
| DM, n (%) | 3 (10.7%) | 3 (42.9%) | 0.079 |
| HTN, n (%) | 14 (50.0%) | 4 (57.1%) | 0.534 |
| Serum creatinine (mg/dl) | 0.84±0.19 | 1.87±0.57 | 0.000 |
| UPCR (g/g Cr) | 4.74±4.51 | 3.55±2.93 | 0.513 |
| GDF-15 (≥ 2.15 ng/ml), n (%) | 6 (21.4%) | 6 (85.7%) | 0.003 |
| PLA_2_R Ab (≥ 2.77 ng/ml), n (%) | 9 (32.1%) | 4 (66.7%) | 0.133 |
| Sclerosis (**≥** 25%), n (%) | 8 (34.8%) | 4 (33.3%) | 0.618 |
| IF/TA (≥ 15%), n (%) | 11 (39.3%) | 6 (85.7%) | 0.036 |

Abbreviations: eGFR, estimated glomerular filtration rate; DM, diabetes mellitus; HTN, hypertension; UPCR, spot urinary protein-to-creatinine ratio; GDF, growth differentiation factor; PLA_2_R Ab; phospholipase A_2_ receptor antibody; IF/TA, interstitial fibrosis/tubular atrophy.

**Supplement Table 3.** Clinical characteristics by interstitial fibrosis/tubular atrophy status

| **Variables (N = 35)** | **Interstitial fibrosis/Tubular atrophy** | | ***P* value** |
| --- | --- | --- | --- |
|  | **(< 15 %, N = 19)** | **(≥ 15 %, N = 16)** |  |
| Age (years) | 56.6±14.1 | 64.0±11.2 | 0.099 |
| DM, n (%) | 2 (10.5 %) | 4 (25.0 %) | 0.248 |
| HTN, n (%) | 9 (47.4 %) | 9 (56.3 %) | 0.427 |
| Serum creatinine (mg/dl) | 0.83±0.18 | 1.31±0.65 | 0.011 |
| eGFR (ml/min per 1.73m^2^) | 95.6±22.3 | 69.4±29.6 | 0.005 |
| Slope of ΔeGFR during 6 months | 0.002±0.045 | -0.025±0.085 | 0.246 |
| UPCR (g/g Cr) | 3.33±3.16 | 5.75±4.91 | 0.411 |
| GDF-15 (ng/ml) | 1.45±0.72 | 2.06±0.72 | 0.016 |
| PLA_2_R Ab (ng/ml) | 2.22±0.72 | 3.43±1.23 | 0.001 |
| Glomerular sclerosis (%) | 13.8±14.2 | 28.2±27.0 | 0.051 |

Abbreviations: DM, diabetes mellitus; HTN, hypertension; eGFR, estimated glomerular filtration rate; Slope of ΔeGFR, (final eGFR – initial eGFR)/6; UPCR, spot urinary protein-to-creatinine ratio; GDF, growth differentiation factor; PLA_2_R Ab; phospholipase A_2_ receptor antibody.
